# Proton Conductive, Low Methanol Crossover Cellulose-Based Membranes

**DOI:** 10.3390/membranes11070539

**Published:** 2021-07-16

**Authors:** Jamaliah Aburabie, Boor Lalia, Raed Hashaikeh

**Affiliations:** NYUAD Water Research Center, Department of Engineering, New York University Abu Dhabi, Abu Dhabi P.O. Box 129188, United Arab Emirates; jha7518@nyu.edu (J.A.); bl83@nyu.edu (B.L.)

**Keywords:** sulfated cellulose, fuel cells, proton exchange membranes, crosslinking

## Abstract

This work describes the development of sulfated cellulose (SC) polymer and explores its potential as an electrolyte-membrane for direct methanol fuel cells (DMFC). The fabrication of our membranes was initiated by the preparation of the novel sulfated cellulose solution via controlled acid hydrolysis of microcrystalline cellulose (MCC). Ion-conductive crosslinked SC membranes were prepared following a chemical crosslinking reaction. SC solution was chemically crosslinked with glutaraldehyde (GA) and cured at 30 °C to produce the aforementioned membranes. Effects of GA concentration on methanol permeability, proton conductivity, water uptake and thermal stabilities were investigated. The crosslinking reaction is confirmed by FTIR technique where a bond between the primary OH groups of cellulose and the GA aldehyde groups was achieved, leading to the increased hydrophobic backbone domains in the membrane. The results show that the time of crosslinking reaction highly affects the proton conduction and methanol permeability. The proton conductivity and methanol crossover (3M) of our GA crosslinked SC membranes are 3.7 × 10^−2^ mS cm^−1^ and 8.2 × 10^−9^ cm^2^ s^−1^, respectively. Crosslinked sulfated cellulose films have lower ion conductivity than the state-of-the-art Nafion (10.2 mS cm^−1^); however, the methanol crossover is three orders of magnitude lower than Nafion membranes (1.0 × 10^−5^ cm^2^ s^−1^ at 1 M). Such biofilms with high methanol resistivity address the major hurdle that prevents the widespread applications of direct alcohol fuel cells.

## 1. Introduction

One of the tremendous challenges of the 21st century is the depletion of energy resources due to growing consumption over the years. The primary source of energy originates from exhaustible fossil fuels such as petroleum. The world consumption of petroleum witnessed an increasing trend over the last half-century, and is expected to continue growing [1]. With this growth, it is predicted that the oil sources and the natural gas reserves discovered so far will soon be diminished [2]. The environmental damage emerging from such sources, such as pollution and global warming, presents additional hurdles. Consequently, much of the efforts are focused and committed to exploring alternative sources of energy. As an example, fuel cells are capable of cleanly converting chemical energy into electrical energy. By combining the best features of engines and batteries, fuel cells are considered attractive for electricity production owing to their high efficiency, compact size, and eco-friendly properties [3]. Fuel cell devices are usually categorized by the kind of electrolyte used in their working arrangements. One type of fuel cells, where a polymer film is used as a proton exchange membrane between the electrodes, is called polymer electrolyte membrane (PEM) fuel cell. PEM fuel cells are constructed using a polymer electrolyte ion-exchange membrane as a proton conductor [4]. PEM fuel cell performance is significantly connected to the properties of the proton exchange membrane used [5]. Direct methanol fuel cells (DMFC) are one of the subcategories of PEM fuel cells where a low-temperature operating system employs methanol as a fuel directly. The noteworthy features of low operating temperature, cost-effective, easy to produce fuel [6], and possible low weight design makes DMFC fuel cells a promising candidate for portable power sources applications [7]. Fabricating membranes for DMFCs with excellent proton conductivity and high resistivity to methanol crossover has been the center of attention of many reports [8,9,10]. 

Nafion^®^, a cation-exchange perfluorinated membrane produced by DuPont, is a standard polymer electrolyte and the benchmark for PEM material for fuel cells. Nafion acquires a phase-segregated unique nanostructure where hydrophobic domain and interconnected hydrophilic ionic channels coexist [11,12]. It is one of the most exploited membranes for DMFC by virtue of excellent proton conductivity and outstanding chemical and thermal stability. In addition, Nafion can be successfully blended and/or modified with other polymers for enhanced performance [13,14,15,16,17]. The major shortcoming of Nafion-based DMFCs is the high methanol crossover, many advances have been proposed to improve the performance of Nafion as a fuel cell polymer electrolyte such as crosslinking, acid treatment, electrical field treatment, and ultraviolet curing [18]. One widely investigated route to mitigating the crossover of methanol in DMFCs is mixed matrix membranes where polymers are infused with inorganic fillers that are decorated with sulfonate or hydroxyl functional groups [19,20,21,22]. Despite the advantageous features of Nafion, there is increasing demand that emerges from the growing number of publications on substituting Nafion with natural polymers owing to their attractive characteristics [23]. The cost of production of natural polymers is notably lower than synthetic polymers on account of their availability in nature. Furthermore, the prolonged degradation period of synthetic polymers will harmfully affect the environment. 

Cellulose is a well-known biopolymer due to its abundance, low cost, excellent hydrophilicity, biocompatibility, variety of possible functionalities, and resistivity to many organic solvents. These captivating features are best explained by the molecular structure of cellulose. Cellulose is a regular homopolymer of d-glucopyranose subunits with exclusively β-1,4-glycosidic linkages; this combination enriches the structure with hydroxyl groups. The hydroxyl group’s vigorous inter and intrachain interactions (hydrogen bonding) are the reason for the structure to be rigid, closely packed, and exceedingly complicated [24]. Cellulose has been widely investigated as a potential electrolyte membrane for DMFC [25], either in its pristine form, blended with other polymers [26], or chemically modified [27]. For example, introducing a proton-donating acid groups method was followed by crosslinking cellulose fibers with sulfonic acid linkers to enhance the proton conductivity [28]. Seo et al. reported the crosslinking of microcrystalline cellulose with sulfosuccinic acid, they succussed in improving the ionic conductivity of the prepared membranes reaching 40.0 mS cm^−1^ [29]. Lin et al. grafted 2-acrylamido-2-methyl-1-propane sulfonic acid (AMPS) using UV-light induced polymerization on bacterial cellulose. Membranes fabricated with 39.5 wt% grafting degrees reported a corresponding proton conductivity in the fully hydrated state of ∼29.0 mS cm^−1^ [30]. Additionally, Guccini et al. reported the utilization of carboxylated cellulose nanofibers (CNFs) as a membrane for DMFC. The preparation was initiated with TEMPO-mediated oxidation of cellulose pulp followed by sodium ion exchange with protons in sulfuric acid. The proton conductivity was estimated at 30 °C and 95% RH to be ∼1.3 mS cm^−1^ [31]. The complexity of the cellulose structure limits its solubility in many organic solvents, which is an important step for processing cellulose into films. A limited range of solvents or solvent systems were explored for fabricating cellulose membranes so far. Among the popular ones, sodium hydroxide/urea systems, ionic liquids, and deep eutectic solvents [32,33,34]. One approach to overcome the challenging solubility is to convert the cellulose to one of its easily processed derivatives, followed by a regeneration step that will revert the derivative to its pristine form [35]. 

Acid-hydrolysis of cellulose is a typical approach to break down cellulose chains; the process is carried out either in dilute or concentrated acid. Cellulose derivatives can be obtained by changing the conditions of the hydrolysis, such as acid concentration, hydrolysis temperature, or regenerating solvent [36]. Sulfated cellulose derivatives [37] are attractive materials owing to their water solubility, ionic conductive features, and antimicrobial properties [38]. High sulfation degree techniques were disclosed previously, such as employing deep eutectic solvents [39]. Films of sulfated cellulose have various applications, such as desalination membranes [40]. Facile technique to prepare sulfated cellulose was previously reported via controlled acid-hydrolysis of microcrystalline cellulose (MCC) using sulfuric acid solution with optimized concentration [41]. Crosslinking of polymers is a well-established method to improve the chemical, thermal and mechanical properties. Crosslinked cellulose was previously investigated to prepare hydrogels [42], nanofiltration membranes [43], and optically transparent films [44,45,46]. Glutaraldehyde (GA) is one of the crosslinking agents that was heavily studied for the crosslinking of cellulose; the reason is that dialdehydes achieve resilience, improve mechanical properties, and impart thermal stability [47]. Several forms of the glutaraldehyde agent can be present in equilibrium in the reagent solution at a given pH; this multicomponent nature is the main reason behind its efficiency as a crosslinking agent [48]. The solubility of GA in water facilitates crosslinking in aqueous media, which is considered a safe and economical approach [49]. Herein, we report the successful preparation of ion conductive sulfated cellulose membranes for DMFCs via a combination of controlled acid-hydrolysis of cellulose and GA-crosslinking reaction. The prepared membranes acquire high chemical and thermal stability with minimal methanol permeability. 

## 2. Materials and Methods

### 2.1. Materials

Microcrystalline cellulose (MCC) powder was supplied by FMC BioPolymer (Avicel-PH101, Philadelphia, PA, USA), 95% Sulfuric acid (H_2_SO_4_) Reagent Grade was procured from VWR Chemicals (Randor, PA, USA), 25 wt.% glutaraldehyde aqueous solution was purchased from Sigma-Aldrich (St. Louis, MO, USA).

### 2.2. Preparation of Sulfated Cellulose Solution 

Sulfated cellulose polymer was prepared following a procedure reported previously [41]. MCC powder was mixed at 35 °C with 70% sulfuric acid solution with a ratio of 10 g MCC to 100 mL acid using Copley Dissolution Tester DIS 6000 (Moschato, Greece) at 200 rpm agitation for 60 min. After mixing, DI water was added to dilute the solution (every 100 mL acid requires 900 mL DI water), which was later centrifuged at 4200 rpm using Thermo Scientific™ (Goteborg, Sweden) multifuge X3R/Heraeus Centrifuge for 10 min. The residual undissolved MCC was disposed and the upper layer was dialyzed under running tab water in a Spectra/Por (MWCO: 12–14 kD) dialysis membrane until the pH of the solution reached 7–8 (around three days). Further dialysis was carried out again, however, with distilled water to reach a pH of 4–5, in efforts to remove all the cations. After dialysis, the yield and the solution concentration were calculated by freeze-drying a known weight and volume of the sample using Labconco™ (Kansas City, MO, USA) freeZone 12L-84C freeze dryer. A 0.5 wt.% SC solution was obtained with a 46% yield. Elemental analysis of the SC material was carried out using CHNS Elemental Analyzer (Elementar-Vario Micro Cube, Langenselbold, Germany). The samples were freeze-dried overnight prior to the analysis, and an average of three runs was reported for the C, H and S percentages.

### 2.3. Fabrication of Crosslinked Sulfated Cellulose Electrolyte Membranes

A 35 g cellulose solution (0.5 wt.%) was crosslinked at room temperature with 3, 6, 9 and 15 g glutaraldehyde (25 wt.% aqueous solution) to prepare a 5%, 10%, 15% and 25% crosslinker concentration, the reaction was catalyzed by the addition of 300 µL of 20% H_2_SO_4_ (to reach a pH of around 1). The final solution was kept at 50 g total. After a reaction time of 3, 6 or 12 h, the mixture was poured into a flat glass plate and left to cure/dry in an environmental oven at 30 °C and humidity level of 10% for 3 h. After drying, the films were immersed in DI water to provoke solidification and detachment followed by washing repeatedly with water to remove any unreactive excess crosslinker.

### 2.4. Membrane Characterization 

#### 2.4.1. Characterization of Morphology and Structure

The Molecular weight of the prepared sulfated cellulose (SC) was measured using Malvern™ (Malvern, UK) ZEN3600 ZetaSizer. Fourier transform infrared (FTIR) (Thermo scientific™, Nicolet iS5 with iD7 ATR accessory) spectrometer with the scanning wavelength range of 4000–500 cm^−1^ was used to characterize the SC pellets and CSC films. X-ray Powder Diffraction (Malvern™, Empyrean 2, Malvern, UK) was used to obtain XRD curves for the freeze-dried samples. Microstructure images were obtained using ThermoFischer™ (Waltham, MA, USA) TAlos F200X Transmission Electron Microscopy (TEM). Samples were prepared by depositing a drop of diluted cellulose suspension on agar scientific^®^ holey carbon S147-4 grids and allowing it to air-dry. Scanning electron microscope (SEM) images were obtained using the ThermoFischer™ Quanta 450 FEG. 

#### 2.4.2. Thermal Stability

The thermal properties of SC powder and CSC films were measured using thermogravimetric analysis (TGA) (Netzsch TG 209 F3 Tarsus, Wunsiedel, Germany). The temperature range employed is 25–1000 °C with a heating rate of 20 °C/min. 

#### 2.4.3. Mechanical Stability

Mechanical analysis of film samples was carried out using a tensile tester (Universal Testing System—Instron 5960, Norwood, MA, USA) with a 50 N load cell at 0.2 mm/min at room temperature, following the ASTM D638-03 standard procedure. The samples were cut in rectangular shape and had a width of 10 mm and a length of 30 mm. 

#### 2.4.4. Contact Angle and Water/Methanol Uptake

Contact angle measurements was performed using an EasyDrop Standard drop shape analysis (DSA100, Krüss GmbH, Hamburg, Germany). DI water droplet of 2 µL in volume was placed on the membrane surface using a 0.51 mm diameter needle syringe. The water uptake% (WU) of the membranes was calculated by observing weight changes between the dry and water-wet state at 25 °C. Initially, membranes at room temperature were submersed in water. After 24 h, membranes were taken from the water and were gently wiped from excess droplets, the wet-weight of the membranes was quickly recorded. The same membranes were then dried at 50 °C for 24 h in a vacuum oven and weighed immediately to obtain the dry weight values. The WU% of membranes were calculated using Equation (1):

WU = ((W_wet, water_ − W_Dry_)/W_Dry_) × 100% (1)

weights of the wet (in water) are referred to as W_wet, water_ and weights of the dried membranes are referred to as W_dry_.

The methanol uptake% (MU) of membranes was similarly studied by recording the changes in the weight of dried and methanol-wet state after membranes immersion in methanol 3M solution at 25 °C. The MU% of the membranes were calculated using Equation (2):

MU = ((W_Wet, methanol_ − W_Dry_)/W_Dry_) × 100% (2)

where W_wet, methanol_ and W_dry_ are weights of wet (in methanol) and dried membranes, respectively.

#### 2.4.5. Ionic Conductivity

An AC impedance method was employed to measure the ionic conductivity of the membranes at a frequency range from 100 kHz to 1 mHz using Autolab 302N Potentiostat/Galvanostat frequency response analyzer, following procedure described previously [50]. A Swagelok-type two electrode cell consists of stainless-steel electrode and membrane surface area of 1 cm^2^ was used. The membranes were used in wet condition at 25 °C. The Nyquist plots were drawn and the conductivity (σ) was calculated by Equation (3):

σ = (G × L)/A(3)

where L represents the thickness, A represents the area of the membrane sample, and G is conductance determined from Nyquist plots. 

#### 2.4.6. Methanol Permeability

A two compartments glass diffusion cell (H-type) was used to observe the permeability of methanol (3M) following a description reported in the literature [51]. The membrane is fixed between two-glass compartments with identical volume. Before starting the permeability test, membranes were washed thoroughly with DI water. Total organic carbon (TOC) analyzer (Sievers InnovOx ES laboratory TOC Analyzer, Trevose, PA, USA) was used to monitor the concentration of methanol in the DI-water compartment. Before the measurements, TOC analyzer was calibrated using three standard concentrations of methanol. Calculated methanol permeability values were obtained following Equation (4):

P = ((dC_b_/dt) × V_b_ × L)/(C_ai_ × A) (4)

where dC_b_/dt is the concentration change with time of methanol in the DI-water compartment; C_ai_ is the initial methanol concentration in the methanol compartment; V_b_ is the DI-water volume in the water compartment; L (cm) is the thickness; and A (cm^2^) is the area of the membrane. 

## 3. Results

### 3.1. Crosslinked Membrane Structure and Morphology Characterization:

**Figure 1:**
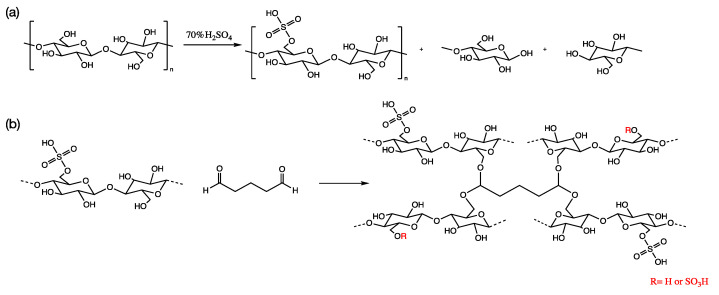
(**a**) Sulfated cellulose preparation via hydrolysis. (**b**) Chemical crosslinking reaction of cellulose with glutaraldehyde.

**Figure 2:**
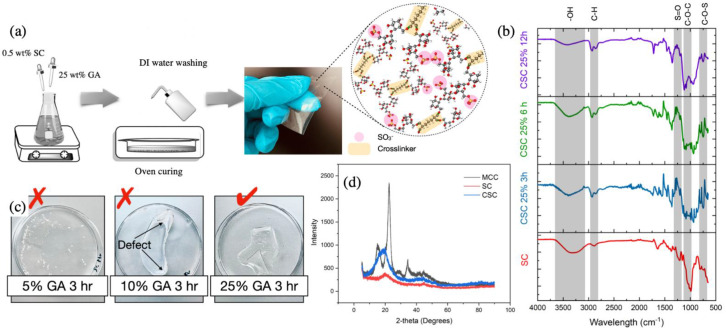
(**a**) Electrolyte membranes preparation procedure, (**b**) FTIR analysis of sulfated cellulose and crosslinked sulfated cellulose with different reaction time, (**c**) digital image showing the films of crosslinked sulfated cellulose with different crosslinker concentration, and (**d**) XRD analysis of MCC, SC, and crosslinked SC with 25% GA for 12 h.

The sulfuric acid hydrolysis of cellulose causes a breakage of the glucosidic bonds and results in esterification of the hydroxyl groups [52]. The reaction scheme is presented in Figure 1a. After the hydrolysis of the microcrystalline cellulose (MCC), sulfated cellulose with low and high-molecular weights was produced in addition to glucose units. After centrifuge, the water-insoluble high-molecular weight cellulose was precipitated at the bottom layer and decanted whereas the low molecular weight water-soluble sulfated cellulose formed the upper layer. The elemental analysis of the freeze-dried low molecular weight sulfated cellulose (SC) solution measured an average percentage of C = 32.66%, H = 5.91%, and S = 3.31%, which confirms a high degree of sulfation. The average molecular weight of the SC was found to be 4.24 ± 0.169 kD. The sulfated cellulose crosslinking reaction with glutaraldehyde is presented in Figure 1b and the preparation procedure is displayed in Figure 2a. Crosslinking occurred between the dialdehyde groups of GA and the primary hydroxyl groups of cellulose (at any position) to form an acetal linkage. This reaction takes place in acidic conditions (in the presence of dilute H_2_SO_4_). The crosslinking reaction is followed by a drying and curing step in a flat glass plate at 30 °C in oven with 10% relative humidity, the curing step guarantees improved crosslinking efficiency. Films were allowed to relax after oven curing and then immersed in water for easy detachment. The excess crosslinker was removed by washing the membranes with DI water repeatedly.

The crosslinker concentration effect on the membrane formation was studied briefly, 5% GA was insufficient to crosslink the SC, 10% GA was leading to form a weak and unstable film as shown in Figure 2c, and crosslinking with 15% and 25% GA forms a stable free-standing membrane. Fourier transform infrared (FTIR) spectroscopy is a reliable method for observing the functional groups of cellulose chains before and after crosslinking. As shown in Figure 2b, the FTIR spectra of SC, which was obtained by shaping the SC material into pellets, is compared to the 25% GA crosslinked sulfated cellulose (CSC) with a different crosslinking reaction time. The peaks of absorption at around 3000–3500, 2750–3000, and 1000–1200 cm^−1^ refer to the OH groups, C−H stretching, and C−O−H and C−O−C asymmetric stretching, respectively. Absorption bands at 1260 cm^−1^ and 814 cm^−1^ are attributed to the stretching vibration of S=O and C-O-S, respectively, as reported previously [53]. A reduced trend of absorption of OH (3000–3500 cm^−1^) of CSC compared to SC was observed by increasing the crosslinking reaction time, suggesting successful crosslinking bonds. The absorption peak at 1115 cm^−1^ is highly evident in the crosslinked film compared to the pristine SC, which is allocated to the ether bond formed between cellulose and GA [40]. After 12 h of crosslinking, the later peak seems to split and increase in intensity suggesting high crosslinking degree. The –OH groups being consumed and the –C–O–C groups increased in intensity with increasing extent of the crosslinking reaction are signs of the crosslinking progress. 

X-ray powder diffraction of the MCC, SC, and the CSC are shown in Figure 2d. Crystalline cellulose I pattern was observed in the XRD curve of MCC, the characteristic peaks at 2θ = 14.7°, 16.5°, and 22.6° are corresponding to the diffraction planes of (−110), (110), and (200), respectively [54]. After the acid hydrolysis and the esterification of MCC, the regeneration of cellulose by water is responsible for the formation of amorphous SC with a peak at 21.9°. We believe that the amorphous structure occurred as a result of the inter- and intramolecular hydrogen bonds between cellulose molecule being rapidly broken during the hydrolysis, leading to destruction of the mother crystalline form. The change from crystalline to amorphous phase is common and reported previously [55,56]. After crosslinking, the material kept the amorphous structure, indicating no significant influence on the crystal patterns by the crosslink reaction [57]. However, the characteristic peak was intensified, possibly due to the stacking formation upon crosslinking. In addition, an increase in the amorphous region-order has been achieved, and improved arrangements of cellulose chains have been established.

**Figure 3:**
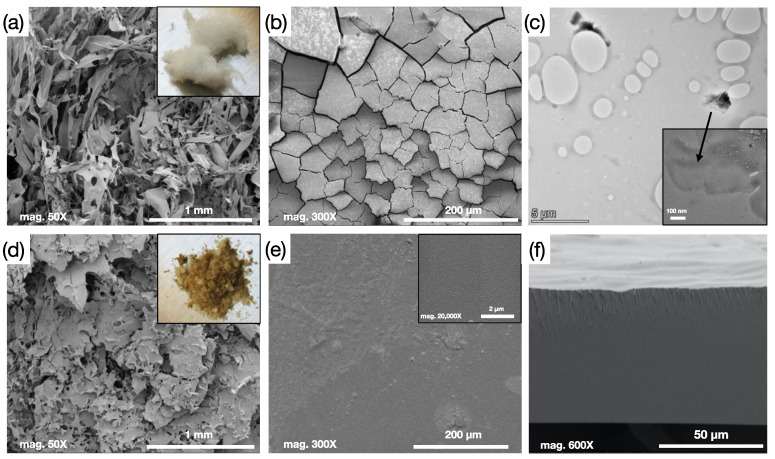
(**a**,**b**) SEM images of the SC material and film with image (**a**) having an inset of digital photo of the powder. (**c**) TEM images of the SC material, inset is a higher magnification image. (**d**,**e**) SEM images of the 3 h CSC with 25% GA material and film with (**d**) image having an inset of digital photo of the crosslinked powder and (**e**) image having a higher magnification inset. (**f**) Cross-section image of the CSC 25% for 3 h membrane displaying dense structure.

SEM images of the freeze-dried sulfated cellulose and solution casted film are shown in Figure 3a,b. Freeze dried sulfated cellulose material has a fibrous-like structure as shown in Figure 3a, and the digital image of the material is presented in the inset. The film obtained after the solution casting of sulfated cellulose has a cracked structure and does not form a continuous film, as can be seen from Figure 3b. Figure 3d,e represents the SEM images of the crosslinked sulfated cellulose and solution casted film. After cross linking, the SC material looks more like a powder and lost its foam-like appearance (inset of Figure 3a) and solution casting formed a homogenous dense film (Figure 3e). Cross-sectional image of the crosslinked membrane is presented in Figure 3f, where a dense morphology is revealed. TEM image of the freeze-dried SC material is presented in Figure 3c. The SC solution was diluted even further and precipitated on the grid to ensure good imaging. SC material was sensitive to beam, and damaging of the sample was evident with high magnification. The image confirms a thin flake structure of SC. During the acid hydrolysis, the MCC cellulose chains were broken into short segments and regeneration of the hydrolyzed cellulose with water resulted in forming short length, low molecular weight, water soluble sulfated chains as a result of the quick precipitation.

### 3.2. Thermal/Mechanical Stability and Contact Angle Measurement

**Figure 4:**
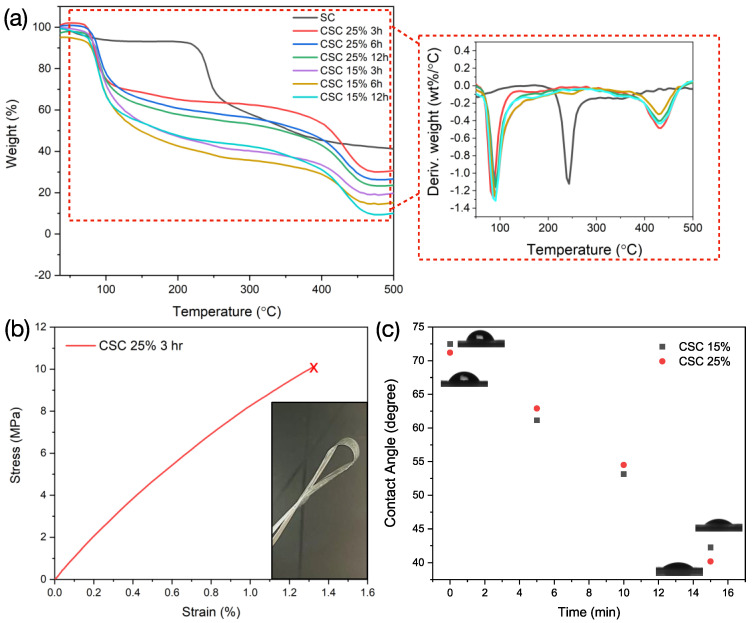
(**a**) TGA curves of SC and CSC membranes, the inset highlights the DTG curves. (**b**) Stress–strain curve for the 25% GA CSC for 3 h, test was repeated with three samples, the inset shows a digital photo of the flexibility of the prepared CSC films. (**c**) Variation of static contact angle with time using deionized water for both CSC films with 25% and 15% GA crosslinked for 3 h.

Thermal stability of sulfated cellulose and crosslinked sulfated cellulose samples was studied using thermogravimetric analysis, and thermograms are shown in Figure 4a. The maximum thermal weight loss reported previously for Microcrystalline cellulose (MCC), which is the starting material of SC, took place at 348 °C [58]. The thermal degradation behavior of the SC polymer showed a lower degradation temperature compared to the mother polymer. The DTG curves revealed that the maximum weight loss was triggered at a temperature of 210 °C for SC. It was previously reported that acid-hydrolyzed bacterial cellulose experienced such degradation behavior (250–300 °C) concluding that with higher sulfate content, degradation shifts to lower temperatures [59]. We believe that the 210 °C degradation is a sign of both sulfate content and decomposition of free-end chains that are formed during the acid hydrolysis [60]. In addition, the sulfate group can act as flame hindering groups and cause an increase in the char fraction; in fact, thermal degradation studies on sulfated cellulose previously reported that sulfation by sulfuric acid leads to lower degradation temperatures [59]. The amorphous content increase in the SC material can also cause the decrease in thermal stability; this is evidenced from XRD data in Figure 2d. Paying close attention, the crosslinked films exhibit peaks at about 100 °C, which is attributed to the evaporation of water adsorbed (that is directly influenced by the amorphous nature of the CSC). All of the crosslinked SC films showcase a peak at a higher degradation temperature (around 390 °C), which indicates higher thermal stability feature is introduced by the crosslinker bonds. From FTIR and TGA analysis, we choose the optimum condition of 25% GA concentration and a crosslinking time of 3 h. 

The stress−strain curve of CSC 25% 3h is shown in Figure 4b. Comparison with the regenerated SC material was not possible, as SC cannot form a homogenous film. The crosslinked film revealed a tensile strength and stress at break of 10 MPa and 1.3%, respectively. GA affected significant the mechanical properties of crosslinked sulfated cellulose films where films exhibited good integrated mechanical property. Similar behavior of the mechanical property improvement of crosslinked cellulose nanofibers was reported in previous research [61]. We believe that with increasing the cellulose concentration as well as increasing the crosslinking time, further enhance of the mechanical properties will be expected.

Crosslinking of the sulfated cellulose imparts increased hydrophobicity to the membrane structure. To check the influence of crosslinking on the hydrophilicity of the membrane, crosslinked samples using 15% and 25% GA for 3 h were tested for the static contact angle of DI water on the membrane surface with time. The results are illustrated in Figure 4c. Static contact angle, at time t = 0 min, showed a value of 72.4° ± 1 and 71.2° ± 1 for crosslinked membrane using 15% and 25% GA, respectively. The static contact angle was measured at different time intervals and showed a linear decrease in contact angle with time. Results highlight similar behavior in terms of water-contact angle for crosslinked membranes regardless of the concentration of GA. 

### 3.3. Water/Methanol Uptake, Proton Conductivity and Methanol Crossover

**Figure 5:**
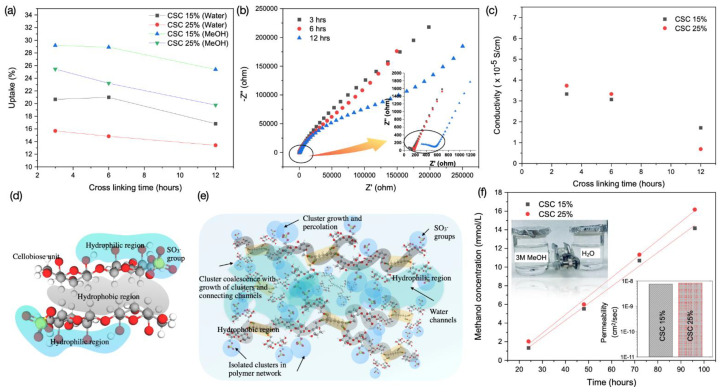
(**a**) Water and methanol uptake of the 15% and 25% crosslinked sulfated cellulose for 3, 6 and 12 h. (**b**) A typical Nyquist plot of the membrane crosslinked with 25% GA for different crosslinking time (3, 6 and 12 h). (**c**) Variation of ionic conductivity with crosslinking time for membrane crosslinked using 15% and 25% GA. (**d**) Hydrophilic (glucopyranose ring plane with hydrogen bonding between the hydroxyl groups) and hydrophobic (glucopyranose ring plane highlighting the backbone hydrogen atoms) parts of cellobiose repeating unit in sulfated cellulose. (**e**) Proposed morphological description for crosslinked sulfated cellulose with cluster-network model. (**f**) Variation of the concentration of methanol (V_b_) in the DI-water compartment (of the diffusion cell shown) with time and the inset present the calculated methanol permeability for the two different membranes using 15% and 25% cross linking agent for 3 h.

The transportation of protons through the membrane is interpreted by either (i) the Grotthuss [62] or (ii) vehicle mechanism [63]. Grotthuss mechanism or so called the hopping mechanism, suggest that the hydrogen bond networks transport protons by allowing OH bonds to be formed and broken in alternative matter. In the vehicle mechanism, on the other hand, protons are carried out by “vehicle molecules”, such as H_2_O, as H_3_O^+^ ions. The water insolubility of cellulose is often explained by the presence and interactions of hydroxyl groups, which forms strong intra- and intermolecular hydrogen bonding network. However, Bergenstrahle et al. demonstrated that aqueous insolubility of cellulose is not caused by hydrogen bonding alone [64,65,66]. In fact, several observations discuss that hydrophobic interactions are crucial to explain the cellulose aqueous insolubility. As a matter of fact, there is a clear partitioning between polar (caused by OH groups) and nonpolar (caused by CH backbone) moieties in the structure of cellulose, and thus a clear amphiphilicity is present [67]. Consequently, a stacking caused by hydrophobic interactions can result in a sheet-like structure showcased in Figure 5d [68]. The SO_3_^−^ group anchored at the sulfated cellulose increase the hydrophilic interaction of the cellulose. Upon solvation (once the film is hydrated), and due to the amphiphilicity nature between the SO_3_^−^ group and the cellulose backbone, a natural phase separation is enhanced. The increased hydrophilicity and the stacked hydrophobicity allow the structure to form channels of water, Figure 5e, similar to the cluster-network model proposed for the perfluorinated polymers like Nafion [12,69]. Due to the crosslinked sulfated cellulose structure, while in wet-state, a phase separation between the hydrophilic and hydrophobic domains occurs, and ionic clusters formation is initiated. The well-connected hydrophilic region and the charged sulfate groups are accountable for the facile proton transport, whereas the crosslinked hydrophobic moiety imparts the mechanical stability. Pristine sulfated cellulose is a water-soluble material owing to the sulfonic group; however, crosslinking with GA resulted in a water-resistant film. 

The utmost importance of water absorption by electrolyte membranes arises from the fact that water acts as a carrier to induce proton conduction [8]; however, the excessive water uptake is considered a downside in many developed membranes, as it will cause swelling and affect the mechanical stability. The wetting capacity of the as-prepared crosslinked sulfated cellulose membranes with 15 and 25% GA for different times (3, 6, and 12 h) were examined by determining the water and methanol uptake, and results are shown in Figure 5a. Water and methanol uptake percentages decrease with increasing crosslinking agent’s concentration. This behavior has been observed due to the increase in hydrophobicity of the crosslinked films as the crosslinker adds more C-H groups to the backbone of the crosslinked cellulose. Additionally, increased GA crosslinker may reduce the size of the hydrophilic channels, resulting in lower free-volume and consequently decreased water uptake. This behavior was previously investigated with sulfosuccinic acid crosslinked polyvinyl alcohol (PV) [70]. For 25% CSC membrane with crosslinking time of 3 h, a water uptake of 15.5% and methanol uptake of 25.5% were recorded, revealing impressive water uptake and resistivity to methanol as a comparison to Nafion membranes (water and methanol uptake of 12.5 and 179.7%, respectively) [17]. 

Crosslinking is usually empowering the films to be resistant to solvation and in this case the solvation is water-induced hence increased hydrophobicity upon crosslinking. Increase the crosslinking time also induce additional crosslinking bond formation and as a result more hydrophobic structure which will lead to a reduction in the uptake of water and methanol. Crosslinked sulfated cellulose has SO_3_^−^ group attached to the cellulose backbone, which helps to impart proton conductivity. The effect of GA concentration and crosslinking time on the ionic conductivity has been investigated. The conductivity of the crosslinked sulfated cellulose membranes was measured at 15% and 25% GA and different crosslinking time of 3, 6 and 12 h. The Nyquist plot of the crosslinked sulfated cellulose membranes as well as the variation of the calculated conductivity of the crosslinked sulfated cellulose with 15% and 25% GA at different reaction time intervals are given in Figure 5b,c, respectively. The Nyquist plots were analyzed to learn the membrane resistance that is used for proton conductivity calculation. The conductivity measurements of the crosslinked membranes showcased the effect of crosslinking reaction time, as the reaction time increased, the conductivity of the membranes seems to drop. For 15% CSC membranes, the conductivity dropped from 3.3 × 10^−5^ S cm^−1^ to 1.7 × 10^−5^ S cm^−1^ at 25 °C by a factor of 1.6 when the reaction time changed from 3 h to 12 h. The conductivity results indicate that higher conductivity favors lower crosslinking reaction time. Similar behavior was noted for membranes crosslinked with 25% GA, where a decrease in conductivity from 3.7 × 10^−5^ S cm^−1^ to 0.69 × 10^−5^ S cm^−1^ at 25 °C by a factor of 3. This is attributed to the additional hydrophobic regions created, which will lower the water wettability that is essential for the proton transfer, and the results are compatible with the water uptake measurement. In addition, the growth of the crosslinked structure will lower the free volume and activate a steric hindrance effect that will suppress H^+^ mobility in water channels, causing the proton conductivity to drop. Furthermore, the higher reticulation caused by the crosslinked network will likely suppress the swelling of the ionic clusters hence reduce the ionic conductivity. Thus, there is a tradeoff between the extent of crosslinking and conductivity of the crosslinked sulfated cellulose membranes. Additionally, for the membranes crosslinked for short time (3–6 h), the conductivity is slightly affected by the concentration of GA. We believe that at that certain time range, the conductivity mechanism is governed by the hopping Grotthuss mechanism. As at this region, the crosslinking intensity is at a minimum and the steric hindrance of the crosslinked structure is interfering with the proton transfer as the ionic exchange sites for proton hopping (sulfonic groups) are hard to reach [71]. However, at extended time of crosslinking (12 h), the crosslinked chains progress and growth creates larger stacked hydrophobic regions that will restrict the water uptake and this will reflect on the proton conductivity, in this case the vehicle mechanism will be predominant. The conductivity results concluded that higher concentration of GA (25%) and lower reaction time (3 h) are optimum reaction condition to obtain a CSC membrane with optimal performance. In comparison with other cellulose electrolyte membranes, CSC membranes reports lower conductivity values than for example cellulose modified with sulfosuccinic acid, which reported a conductivity of 23.0 mS cm^−1^ [29], cellulose nanofibers (0.01 mS cm^−1^ at 100% RH) and cellulose nanocrystals (0.7 mS cm^−1^ at 100% RH) [72]. The conditions chosen here were optimized to reach a combination of good membrane formation, good mechanical properties and acceptable conductivities.

Methanol crossover is a crucial parameter for polymer membranes used in DMFCs. The methanol permeation from the anode, through the membrane to the cathode will lower the voltage and lessen the efficiency of the fuel cell. Hence, extremely low methanol crossover is favored to post up the performance of the fuel cell. To demonstrate the methanol permeability of sulfated cellulose membranes, two compartment H-type glass diffusion cells were used (as shown in inset of Figure 5f). One compartment of the diffusion cell was filled with 3M methanol solution while DI water was placed in the other compartment, these two compartments were separated by the CSC membrane of active surface area of 1.766 cm^2^. Permeation of methanol through the membrane was monitored with time by recording the change of methanol concentration in the DI compartment. The change in methanol concentration in the water compartment against time is presented in Figure 5f. Calculated methanol permeability of the CSC 15% and 25% are presented in the inset of Figure 5f. Sulfated cellulose membrane crosslinked with 25% GA allowed a slightly higher resistance towards methanol permeation compared to membrane crosslinked with 15%. The permeability results demonstrate that the methanol crossover of the 25% crosslinked sulfated cellulose membrane (8.28 × 10^−9^ cm^2^ s^−1^ at 3M) is three orders of magnitude lower than that of Nafion membrane (1.0 × 10^−5^ cm^2^ s^−1^ at 1M) reported in the literature [72,73]. Sulfonated polyethersulfone (PES) was previously reported as nonfluorinated polymer candidate for DMFC with low methanol (4M) permeability of 1 × 10^−10^ cm^2^ s^−1^ at 30 °C [74].

Ionic conductivity and methanol crossover are essential parameters for a successful DMFC. Although high conductivity alongside low methanol permeability is the ultimate goal, trade-off between the two is difficult to achieve. In spite of the low proton conductivity achieved by CSC membrane in this study (3.7 × 10^−2^ mS cm^−1^) in comparison with Nafion (37.5 mS cm^−1^ at 30 °C) [17] and sulfonated PES (1.5 mS cm^−1^ at 30 °C) [74], the extremely low methanol crossover reveals a good potential for such membranes. Improving the ionic conductivity of CSC membranes is possible as a future work. Reported research that involves DMFC does not allow an easy comparison among the different membranes nor indicates which are the best for DMFC application. The reason behind this is the different conditions such as type of polymer, method of preparation, fillers used, thickness, temperature, and method of ionic conductivity testing. Most of the papers reporting high DMFC performance are associated with perfluorosulfonate polymer (Nafion type) electrolytes.

## 4. Conclusions

We have demonstrated the successful fabrication of crosslinked sulfated cellulose in a two-step process. First the sulfated cellulose (SC) was prepared by acid hydrolysis. The hydrolysis process produces a water-soluble material that has potential applications. Second, the water-soluble sulfated cellulose is made into membranes by the crosslinking of its chains with the use of glutaraldehyde. Upon drying, a highly dense membrane was formed. The crosslinked sulfated cellulose (CSC) membranes exhibited good mechanical stability, allowing the tailoring of films. CSC membranes crosslinked with 25% GA for 3 h exhibit extremely low methanol crossover (8.28 × 10^−9^ cm^2^ s^−1^ at 3M), which is favorable for DMFCs. At the same time, the fabricated membranes were conductive to protons with a conductivity of 3.7 × 10^−2^ mS cm^−1^. The crosslinked membranes have a 15.5% water uptake, which is considered low, as a result of the increased hydrophobicity of the backbone of the cellulose. Thermogravimetric analysis revealed a thermally stable membrane with degradation temperature of 390 °C. Crosslinked sulfated cellulose fabricated here are considered environmentally friendly polymer electrolyte alternative that outperform Nafion in terms of methanol barrier property.

## Data Availability

Not applicable.
